# Mr. Ring on Cathartics in Gout

**Published:** 1811-11

**Authors:** J. Ring


*To the Editors of the Medical and Physical Journal.*

Gentlemen,

In your Journal for August, 1804, is an article on the subject of Gout, detailing the plan pursued, in the treatment of the disorder, by some practitioners in Portugal, written by Dr. Tavares, first physician in that kingdom, and communicated by Dr. Adams, who prefixed an observation, that since the days of Sydenham, physicians seem to be afraid of gout. This assertion is too general, as I have proved in my treatise on that subject, by the authority and example of the most celebrated modern authors. Lister ridiculed Sydenham, and treated his cautions with contempt. Cheyne informs us, that many physicians of his acquaintance showed no respect to Sydenham in such cases; and Mead, how much soever he might venerate his name, most decidedly differed from him in the treatment of the gout.

Dr. Adams says, those who peruse Sydenham with care will find he is by no means chargeable with the practice, which so generally prevailed after his time; that, on the contrary, there are cases in which he advises bleeding; and that it it was his custom occasionally to purge himself. Had Dr. Adams himself read Sydenham with a little more care, he would have found, that though he allows a vein may be opened, in the beginning of a fit, in those who are young, and overheated by hard drinking; yet, with respect to purging, he says, emetics or cathartics will only invite the gouty matter back into the blood; and this has often proved fatal, when had recourse to by way of prevention, or, what is worse, by way of easing pain in the fit.

He tells us he is fully convinced that purging, either in the fit, at its decline, or in an interval, is highly injurious; and only serves to aggravate the disease. He tells us there have been empirics, who have acquired considerable reputation by giving concealed cathartics in the gout; that while these cathartics operate, their patients feel little or no pain; and that by continuing them a few days, the fit may be removed: but the patient will have reason to repent of its removal. He assures us indeed repeatedly, and in the strongest terms possible, that it is equally useless and pernicious to attempt to cure the gout by evacuations.

It is true, in his Dissertation on Bloody Urine, he informs us, that having pain in his kidneys, he took two ounces and half of manna once a week, dissolved in a quart of whey, with a little lemon-juice occasionally, to quicken the operation, and render it agreeable to the stomach; but this was for the bloody urine, and not for the gout. On the contrary he declares, that although he retracts his former assertion, of its being absolutely improper to purge gouty persons, either at the beginning or at the decline, or in the intervals of the fits; yet, with regard to the gout itself, all sorts of evacuations are extremely pernicious in that disorder, and ought not to be used, unless bloody urine, and its concomitant symptoms, require them.

The method of cure described by Dr. Tavares was communicated to him by Professor de Lemos of Coimbro, who being called to see a monk, labouring under a severe paroxysm of the gout, recommended palliatives; but a country surgeon, who was present, smiled at his simplicity, and undertook to cure the patient. His plan consisted of half a dram of resin of jalap, half a dram of scammony, and half an ounce of syrup of poppies; and afterwards, a dram of Peruvian bark every hour. At the next visit which he received from the Professor, he was able to walk on crutches; and in two days he quitted his house.

Here is a medicine, which exactly resembles the Eau Medicinale. Jalap, and many other cathartics, when taken in large doses, often prove emetic; and, when joined with opium, also also possess the sudorific property of the compound powder of Ipecacuanha. This mode of treating the gout, though too rash and hazardous for the Professor implicitly to adopt it, suggested to him the propriety of administering a milder cathartic in arthritic complaints. Accordingly, he gave his patients an ounce, or an ounce and half, of sulphate of magnesia, on the accession of a paroxysm, and afterwards a dram of bark every hour; and his practice was crowned with great success.

Dr. Tavares tried this method in his own case, with equal success; and, finding that the bark, in large doses, commonly purged him, took a scruple of jalap, and eight grains of submuriate of mercury, which hastened the cure. When inflammation was considerable, he applied leeches.

Recollecting that an encomium on bark, as a remedy for the gout, appeared in this Journal, I tried to find the article, but was not so fortunate as to meet with it till my publication was far advanced. I therefore availed myself of what Mr. Parkinson has said on the subject. Mr. Parkinson accounts for the mode of operation by which the gout is relieved, in this case, according to the principles of the humoral pathology; but it is full as easy to account for it according to the modern theory of the medical world. Tavares himself, agreeably to Mr. Parkinson's opinion, but contrary to that of Dr. Kinglake, declares that the notion of the humoral pathology is now almost obsolete.

I am, Gentlemen,

Yours, &c.

J. RING.

